# Effectiveness of integrated yoga therapy in treatment of chronic migraine: randomized controlled trial

**DOI:** 10.1186/1129-2377-14-S1-P116

**Published:** 2013-02-21

**Authors:** N Sharma, S Singhal, AP Singh, CM Sharma

**Affiliations:** 1 NMP Medical Research Institute, India; 2 Sms medical college and hospital, India

## Introduction

Yoga intervention was found significant in reducing the headache activity, medication intake, symptoms, anxiety and depression in migraine without aura.

## Purpose/background/objectives

To evaluate the effectiveness of a integrated yoga therapy program for chronic migraine treatment methods study was conducted in low cost, nonclinical setting, a prospective, randomized, controlled trial was conducted in Jaipur, India. Subjects aged 18 to 65 years with 15 or more headache days per month, at least half of which were migraine/migrainous headaches, were randomized 1:1 to either yoga therapy or standard management. Seventy men and women were randomly assigned. The intervention group went through individualized yoga treatment for 12-weeks with 4 consecutive therapeutic sessions a week. Each therapy session lasting for about 60 minutes focusing on strengthening, relaxation, releasing muscular tension and increasing self efficacy. The control group consisted of standard care with the patient's physician. Outcome measures included self-perceived pain intensity, frequency, and duration; functional status; depression; prescription and nonprescription medication use. Outcomes were measured at the end of the 12-week intervention and at a 6-month follow-up.

## Results

Thirty-one of 35 patients from the intervention group and all 35 patients from the control group completed the study. There were no statistically significant differences between the 2 groups before intervention. Intention to treat analysis revealed that the intervention group experienced statistically significant changes in self-perceived pain frequency (P =.000), pain intensity (P =.001), pain duration (P =.000), functional status (P =.000), medication used (p<0.01) and depression (P =.000); these differences retained their significance at the 6-month follow-up.

## Conclusions

Positive health related outcomes in chronic migraine can be obtained with a low cost, group, integrated yoga in a community based nonclinical setting.

## References

[B1] John PJ Sharma N Sharma CM Kankane A Effectiveness of yoga therapy in the treatment of migraine without aura: a randomized controlled trial Headache 2007 14 5 654 61 10.1111/j.1526-4610.2007.00789.x 17501846

